# MicroRNA-181a Functions as an Oncogene in Gastric Cancer by Targeting Caprin-1

**DOI:** 10.3389/fphar.2018.01565

**Published:** 2019-01-10

**Authors:** Qiang Lu, Yanchun Chen, Dan Sun, Shukun Wang, Kang Ding, Meiyi Liu, Yan Zhang, Yujuan Miao, Huancai Liu, Fenghua Zhou

**Affiliations:** ^1^Department of Pathology, Weifang Medical University, Weifang, China; ^2^Department of Histology and Embryology, Weifang Medical University, Weifang, China; ^3^Neurological Disorders and Regenerative Repair Key Laboratory, Weifang Medical University, Weifang, China; ^4^Department of Bioscience and Technology, Weifang Medical University, Weifang, China; ^5^Department of Joint Surgery, Affiliated Hospital of Weifang Medical University, Weifang, China

**Keywords:** miRNA-181a, oncogene, gastric cancer, caprin-1, metastasis

## Abstract

MicroRNA-181a (miRNA-181a) is a multifaceted miRNA implicated in various cellular processes, particularly in cell fate determination and cellular invasion. It is frequently expressed aberrantly in human tumors and shows opposing functions in different types of cancers. In this study, we found that miRNA-181a is overexpressed in Gastric cancer (GC) tissues. Clinical and pathological analyses revealed that the expression of miRNA-181a is correlated with tumor size, lymph node metastasis, distant metastasis, and TNM stage. Kaplan-Meier analysis indicated that overexpression of miRNA-181a is associated with poor overall survival of patients with GC. Moreover, miRNA-181a is overexpressed in GC cells, and downregulation of miRNA-181a induced cell apoptosis and suppressed the proliferation, invasion, and metastasis of GC cells both *in vitro* and *in vivo*. Target prediction and luciferase reporter assay showed that caprin-1 was a direct target of miRNA-181a. Downregulation of caprin-1 expression resulted in a converse change with miRNA-181a in GC. Spearman’s correlation test confirmed that the expression of miRNA-181a expression was inversely correlated with that of caprin-1 in GC cells. Furthermore, the expression of caprin-1 increased after downregulation of miRNA-181a in the GC cells. Caprin-1 siRNA can rescue the oncogenic effect of miRNA-181a on GC cell proliferation, apoptosis, migration, and invasion. These findings suggest that miRNA-181a directly inhibits caprin-1 and promotes GC development. miRNA-181a could be a target for anticancer drug development.

## Introduction

Gastric cancer (GC) is the fifth cause of cancer incidence and the third cause of cancer-related death worldwide (Kim et al., 2018). Although surgery and chemotherapy have improved considerably in the past few decades, the 5-year survival rates of patients with advanced or metastatic GC have remained low, usually ranging from 5 to 20%, and the median overall survival of the patients is less than 1 year (Docrat et al., 2018). A better understanding of the molecular mechanism underlying tumor progression and metastasis may contribute to the design of novel therapeutic target of GC.

MicroRNAs (miRNAs) are a novel class of 20–22 nt-long small non-coding RNAs that control the stability and translation of target genes (Bellazzo et al., 2018). Thus far, more than 1800 human miRNAs have been identified, and most of them are involved in the regulation of virtually all biological processes (Xia et al., 2015). In tumors, miRNAs affect every step of carcinogenesis, including proliferation, apoptosis, migration, and metastasis (Bracken et al., 2016). Emerging evidence has shown that various miRNAs contribute to the tumorigenesis and progression of GC. For instance, miR-506 suppresses GC angiogenesis and epithelial-to-mesenchymal transition (EMT) by targeting ETS1 (Li et al., 2015). MiR-616-3p promotes angiogenesis and EMT in GC through the PTEN/AKT/mTOR pathway (Wu et al., 2018). MiR-422a expression is downregulated and involved in metabolic reprogramming by PDK2 in GC (He et al., 2018). However, the function of miRNA-181a in GC remains to be validated.

In the present study, we demonstrated that miRNA-181a expression is upregulated and caprin-1 expression is downregulated in GC tissues and cell lines. The ectopic expression of miRNA-181a promoted the proliferation, invasion, and migration of GC cells and inhibited apoptosis *in vitro* and *in vivo*. Furthermore, we identified caprin-1 as the direct target gene of miRNA-181a in GC.

## Materials and Methods

### Patient Samples and Cell Culture

Ninety human GC and thirty adjacent non-tumor tissue samples were obtained from the Affiliated Hospital of Weifang Medical University. The samples were obtained through surgical resection. Pathological diagnosis was evaluated according to the World Health Organization classification system. The samples were classified as adenocarcinoma (*n* = 90). The experiments were approved by the Ethics Committee of the Affiliated Hospital of Weifang Medical University, and all the patients gave written informed consent. The samples were collected in accordance with the approved guidelines. None of the patients received chemotherapy or radiotherapy before sample collection. Four GC cell lines (MKN45, SGC-7901, MGC803, and BGC-823), normal gastric epithelial cell line GES-1, and 293T cells were obtained from the American Type Culture Collection and cultured at 37 °C in DMEM supplemented with 10% fetal bovine serum (Hyclone, United States) in an atmosphere containing 5% CO_2_.

### Quantitative RT-PCR

Total RNA was isolated from the tissue and cell samples with TRIzol reagent (Life Technologies, Carlsbad, CA, United States). The amount of RNA was quantified with an ND-1000 spectrophotometer (Nano-drop 3000c, Thermo Scientific, MA, United States). To detect the mRNA levels of miRNA-181a and caprin-1, we performed qRT-PCR according to the manufacturer’s instructions as previously described (Zhou et al., 2013, 2018). The following primers (Sangon Biotech, China) were used in reverse transcription: (miRNA-181a), 5′-GTCGTATCCAGTGCAGGGTCCGAGGTATTCGCACTGGATACGACACTCAC-3′, (U6), 5′-CGCTTCACGAATTTGCGTGTCAT-3′. The following primers (Sangon Biotech, China) were used for PCR: miRNA-181a sense, 5′-GCGGCGGAACATTCAACGCTGTC-3′, miRNA-181a antisense, 5′-ATCCAGTGCAGGGTCCGAGG-3′; U6 sense, 5′-GCTTCGGCAGCACATATACTAAAAT-3′, U6 antisense, 5′-CGCTTCACGAATTTGCGTGTCAT-3′. Caprin-1 sense, 5′-AGGCTGGGACAAGTAAACCTT-3′, Caprin-1 antisense, 5′-TCATTAGCAGGAGGGACTGG-3′; β-actin sense, 5′-TGACGTGGACATCCGCAAAG-3′, β-actin antisense, 5′-CTGGAAGGTGGACAGCGAGG-3′. Endogenous U6 expression was used as the control for miRNA-181a, whereas endogenous β-actin expression served as the control for caprin-1. The differences in the relative expression levels of miRNA-181a and caprin-1 were calculated using the 2^-ΔΔCt^ method.

### Immunohistochemistry and Evaluation Criteria

The expression of caprin-1 in the human GC samples and adjacent non-tumor tissues were determined by immunohistochemistry (IHC). The tissues were fixed in 4% paraformaldehyde, embedded in paraffin, and sliced into consecutive tissue sections. Then, the tissue sections were deparaffinized, dehydrated, and heated in citrate buffer (pH 6.0) for antigen retrieval. To block the non-specific bindings of the first antibody, we added 1% bovine serum onto the slides for 20 min at 37°C. The tissue sections were then incubated with caprin-1 polyclonal antibody (1:100; Fitzgerald, United Kingdom). Finally, the sections were visualized with a DAB kit (ZSGB-bio, China) and counterstained with hematoxylin (ZSGB-bio, Beijing, China). We omitted primary antibody and added phosphate buffered saline (PBS) to the sections as negative controls. The sections were photographed with an optical microscope (Olympus, Tokyo, Japan) and then analyzed with the Image-ProPlus6.0 analytic system (IPP6.0).

### Western Blot Analysis

Western blot analysis was performed according to a standard method described previously. A polyclonal caprin-1 antibody (1:1000; Fitzgerald) was used. Mouse monoclonal antibody GAPDH (1:2000; Proteintech Group, Chicago) was used as loading control. The density of the caprin-1 protein band was quantified after it was normalized to the density of the GAPDH band in the same sample during Western blot analysis. The procedures were performed in decuplicate.

### Cell Transfection

SGC-7901 cells, which presented the highest miRNA-181a expression level in the four GC cell lines, were transfected with RFP-miRNA-181a (control-miRNA-181a-down or miRNA-181a-down) plasmid (GenechemBiotech, Shanghai, China) or cotransfected with RFP-miRNA-181a plasmid and siRNA-caprin-1 (RIBOBIO, China). The transfection was performed with Lipofectamine 3000 (Life Technologies, Carlsbad, CA, United States) according to the manufacturer’s instructions. Stable transfectants were selected, incubated with 600 μg/mL of G418 after 48 h of transfection. All the procedures were performed in triplicate.

### Reporter Vector Construction and Luciferase Reporter Assay

HEK293T cells were used in the luciferase reporter assay. The putative miRNA-181a binding sites in the 3′UTR of caprin-1 were subcloned into a pMIR-report vector (Ambion, Austin, TX, United States), named caprin-1-WT. The primer sequences used for the amplification of caprin-1 3′-UTR were 5′-GAGGAGTTGTGTTTGTGGAC-3′ (forward) and 5′-GCGAGGTCCGAAGACTCATTT-3′ (reverse). Efficient insertion was confirmed by sequencing. Mutant caprin-1 3′-UTR bearing a substitution of six nucleotides (GAAUGU to CTTTAT) in the miRNA-181a target sequence was named caprin-1-Mut. The MiRNA-181a precursor and negative control (miR-con) were purchased from GenechemBiotech (Shanghai, China). Luciferase activities were determined with Promega dual-luciferase reporter system. All the assays were performed in triplicate.

### Cell Proliferation Assay

Human GC cell proliferation index was measured with Cell Counting Kit-8 (CCK-8) assay (Beyotime Institute of Biotechnology, Jiangsu, China) and 5-ethynyl-2-deoxyuridine (EdU) incorporation assay (RIBOBIO, China) kits according to the manufacturers’ instructions. In the CCK-8 assay, the cells were cultured in a 96-well plate. CCK-8 was added into the medium and incubated at 37°C for 4 h. Absorbance (OD) was measured at 450 nm at 24, 48, 72, and 96 h after transfection. EdU incorporation assay was performed as previously described (Wang et al., 2017). The percentage of EdU-positive cells was calculated from five random fields, and all experiments were repeated five times.

### Cell Apoptosis Assays

The apoptosis of cells was measured with a Cell-Light^TM^ EdUTP Apollo^®^488 TUNEL cell detection kit (RIBOBIO, China). Approximately 5 × 10^4^ cells were seeded into each well of the 96-well plates and was examined 48 h after transfection. Cells were fixed with 4% paraformaldehyde (pH 7.4) for 20 min. After being washed with PBS solution for three times, the samples were incubated with 50 μL of reaction buffer (TdT Enzyme 5 μL+Labeling Safe Buffer 45 μL) for 60 min at 37°C. The labeling procedure was stopped by washing the samples with 2% BSA solution three times. The TUNEL incorporation rate was expressed as the ratio between the EdUTP-positive and total Hoechst 33342-positive cells. The percentage of the EdUTP-positive cells was calculated from five random fields, and all the experiments were repeated five times.

### Transwell Migration and Invasion Assays

Transwell migration and invasion assays were performed according to a standard method (Wang et al., 2017). The images of the invaded or migrated cells were photographed, and the cells were counted in five random fields. The average number of cells were obtained from three independent experiments and quantified by the PPI software (Media Cybernetics, United States).

### *In vivo* Proliferation and Metastasis Assays

A mouse xenograft model was established in 4-week-old male NU/NU nude mice (Beijing HFK Bioscience Co., Ltd, Beijing, China) in accordance with the institutional guidelines. The mice were manipulated and housed according to the protocols approved by the Ethics Committee of Weifang Medical University.

The miRNA-181a-silenced SGC-7901 cells and their parallel control cells were used for the *in vivo* proliferation and metastasis assay. For the cell proliferation assay, 1 × 10^6^ SGC-7901 cells were suspended in 200 μL of PBS and then injected subcutaneously of each nude mice (three mice per group). The tumors were observed every 7 days. The mice were euthanized after 5 weeks, and the subcutaneous tumors were isolated, measured, and calculated with the following formula: length × (width)^2^ × 1/2. The tumors were collected for the detection of miRNA-181a and caprin-1 by qRT-PCR and Western blot analysis. For tumor metastasis assay, 5 × 10^6^ SGC-7901 cells in 200 μL of PBS were injected into the tail veins of the NU/NU nude mice (four for each group). The mice were euthanized after 12 weeks, and the entire lung tissues were isolated. The tissue was cut into sections and then stained with hematoxylin and eosin for micrometastasis detection.

### Statistical Analysis

All the data were presented as means ± standard deviation. Statistical analyses were performed with GraphPad Prism5 (San Diego, CA, United States). The differences in the expression of miRNA-181a and caprin-1 in the GC tissues and GC cell lines were assessed by Student’s *t*-test and analysis of variance. The association of miRNA-181a expression with clinicopathological parameters was analyzed with Chi-squared and Fisher’s exact tests. The correlation between the miRNA-181a and caprin-1 expression was tested by Spearman’s correlation. The Kaplan–Meier method was used for the analysis of the survival curve, and the differences were determined by the log-rank test. *P* < 0.05 was considered statistically significant.

## Results

### Overexpression of miRNA-181a in GC Tissues Was Associated With Poor Patient Survival

**FIGURE 1:**
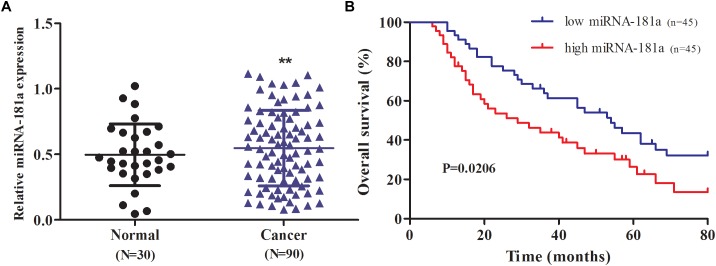
Expression of MicroRNA-181a (miRNA-181a) in human GC tissues and its association with patient survival. **(A)** qRT-PCR on the expression of miRNA-181a in the GC tissues; **(B)** Correlation between miRNA-181a expression and overall survival in patients with GC. *P*-values for Kaplan–Meier curves were calculated with a log-rank test; ^∗^ *P* < 0.05 and ^∗∗^*P* < 0.01.

To define the role of miRNA-181a in GC, we evaluated the expression of miRNA-181a in the GC tissues through qRT-PCR. The expression of miRNA-181a was significantly higher in the GC tissues in contrast to that in the adjacent non-tumor tissues (Figure 1A; *P* < 0.01). To determine the relationship between expression of miRNA-181a and clinicopathological features, we divided the 90 patients with GC into two groups according to their median miRNA-181a level, namely, the high miRNA-181a expression group (above the median level) and low miRNA-181a expression group (below the median level). Clinicopathological analysis revealed that the high expression of miRNA-181a correlates with larger tumor size, more lymph node and distant metastasis, and high TNM stage (Table 1). To further determine the role of miRNA-181a in GC development, we performed follow-ups on all the patients and determined their overall survival after surgery. Kaplan–Meier survival indicated that the overall survival of patients with high miRNA-181a expression was significantly lower than that of low miRNA-181a expression (*P* = 0.0206; Figure 1B).

**Table 1:** Clinicopathologic characteristics of GC associated with miRNA-181a expression.

Characteristics	MiRNA-181a expression		Median	*p*-value
			
	High (Number)	Low (Number)		
**Age(years)**				
≥60	18	34	0.5250	0.2849
<60	11	27	0.5485	
Gender				
male	19	35	0.55	0.4613
female	10	26	0.5215	
**Tumor size(cm)**				
<5	9	34	0.474	0.0283^∗^
>5	20	27	0.631	
**Differentiation status**				
well	4	11	0.474	0.8806
moderate	15	30	0.575	
poor	10	20	0.525	
**TNM stage**				
I+II	8	34	0.399	0.0141^∗^
III+IV	21	27	0.651	
**Lauren’s classification**				
Intestinal	18	32	0.554	0.3912
Diffuse	11	29	0.5345	
**Distant metastasis**				
Negative	22	56	0.512	0.0376^∗^
Positive	7	5	0.762	
**Lymph node metastasis**				
negative	8	34	0.3745	0.0124^∗^
positive	21	27	0.637	


*^∗^*P* <0.05.*

### Down-Regulation of miRNA-181a Inhibited the Proliferation, Migration, Invasion and Enhanced Apoptosis of SGC7901 Cells

**FIGURE 2:**
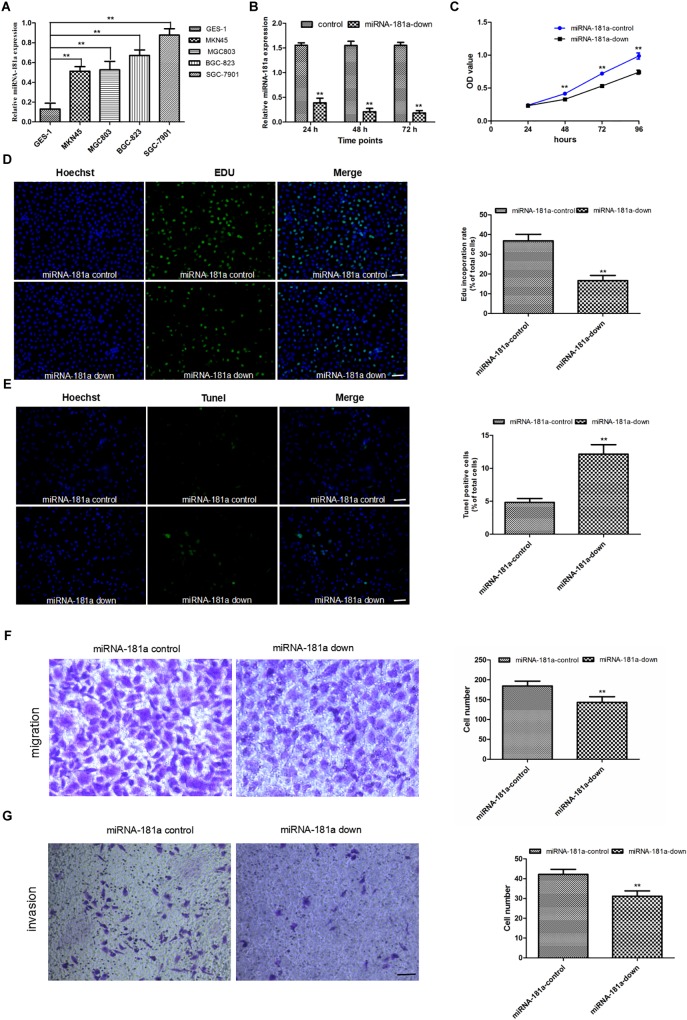
Downregulation of miRNA-181a inhibited the proliferation, invasion, and metastasis, as well as enhanced the apoptosis of the SGC7901 cells. **(A)** MiRNA-181a expression increased in the GC cell lines as detected by qRT-PCR; **(B)** Relative miRNA-181a expression decreased in the SGC7901 cells transfected with siRNA miRNA-181a; **(C–D)** Cell proliferation was inhibited in the miRNA-181a downregulated SGC7901 cells, as indicated by the CCK-8 (24, 48, 72, and 96 h) and EDU (48 h) assay, bar = 100 μm; **(E)** Cell apoptosis was enhanced by EdUTP TUNEL Cell Detection Kit in the SGC7901 cells (48 h), bar = 100 μm; **(F–G)** Transwell migration and invasion were inhibited in the SGC7901 cells (48 h), bar = 50 μm; ^∗^ *P* < 0.05 and ^∗∗^*P* < 0.01.

To confirm the results of the human GC samples, we assessed the miRNA-181a level in GC cells using qRT-PCR. Results showed that miRNA-181a level was higher in the GC cells than in the normal gastric epithelial GES-1 cell. The metastatic GC SGC7901 cell had the highest level of miRNA-181a (Figure 2A). To examine whether miRNA-181a can affect the biological characteristics of GC, we down-regulated the expression of miRNA-181a in the SGC7901 by miRNA-181a-down plasmid transfection. qRT-PCR was performed to assess the transfection efficiency. It was found that the relative expression of miRNA-181a was significantly lower in the miRNA-181a-down plasmid transfected SGC7901 cells than in the negative control group (Figure 2B). The effects of miRNA-181a on cell proliferation and apoptosis were assessed through CCK-8, EDU, and TUNEL incorporation assays. The result indicated that downregulation of miRNA-181a inhibited proliferation and enhanced apoptosis of the SGC7901 cells (Figures 2C–E). To investigate the role of miRNA-181a on the migration and invasion of SGC7901 cells, we performed transwell invasion and migration assays 48 h after transfection with miRNA-181a or negative control. The results showed that downregulation of miRNA-181a inhibited migration and invasion of the SGC7901 cells (Figures 2F,G).

### Downregulation of miRNA-181a Suppressed GC Growth and Lung Metastasis *in vivo*

**FIGURE 3:**
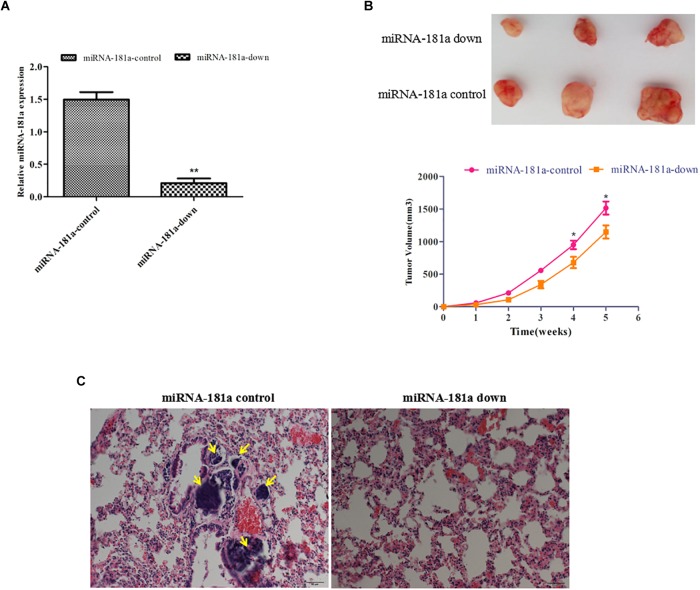
Downregulation of miRNA-181a suppressed GC growth and lung metastasis *in vivo*. **(A)** qRT-PCR on the expression of miRNA-181a; **(B)** The average size of subcutaneous tumors decreased in miRNA-181a downregulated group; **(C)** Metastasis to the lungs observed in the NC group, whereas no metastatic locus was found in the miRNA-181a downregulated group after 12 weeks, bar = 100 μm; ^∗^*P* < 0.05 and ^∗∗^*P* < 0.01.

To determine whether miRNA-181a inhibits growth and metastasis *in vivo*, SGC7901 cells expressing ectopic miRNA-181a were injected subcutaneously or into tail veins of the nude mice. As shown in Figure 3A, tumors of different sizes formed in the left abdominal subcutaneous tissues after 5 weeks. The average size of the tumors decreased, and miRNA-181a expression markedly decreased, as shown by qRT-PCR, in the miRNA-181a downregulated group, in contrast to those in the NC group (Figures 3A,B). Furthermore, tumor metastasis to the lungs was found in two of the four injected mice in the NC group, whereas no metastatic locus was observed in the miRNA-181a downregulated group after 12 weeks (Figure 3C). Overall, downregulation of miRNA-181a significantly inhibited tumor growth and metastasis in the mouse xenografts.

### Caprin-1 Was Downregulated in the GC Tissue and Was a Target of miRNA-181a in GC

**FIGURE 4:**
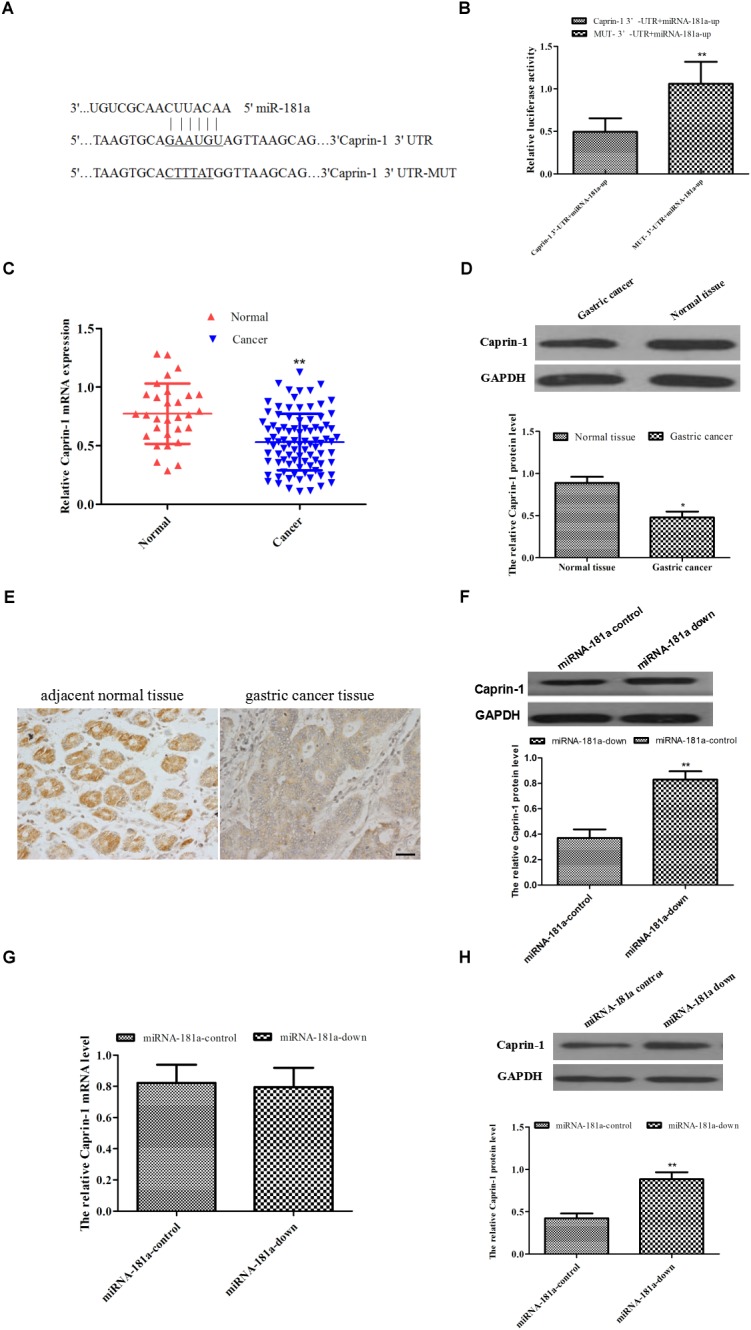
Caprin-1 was downregulated in the GC tissue and was a target of miRNA-181a in GC. **(A)** Binding sites of miRNA-181a to 3′-UTR and mut-3′-UTR of caprin-1 mRNA; **(B)** Luciferase activity of HEK293T cells transfected with miRNA-181a precursor and caprin-1-WT plasmid; **(C)** Caprin-1 mRNA decreased in the GC tissues; **(D)** Caprin-1 protein decreased in the GC tissues; **(E)** IHC analysis on expression of caprin-1, bar = 50 μm; **(F)** Protein of Caprin-1 in the SGC7901 cells after transfection with miRNA-181a downregulated plasmid; **(G)** mRNA level of caprin-1 in the SGC7901 cells after transfection with miRNA-181a downregulated plasmid; **(H)** Caprin-1 protein level increased in the subcutaneous tumors in xenografts; ^∗^*P* < 0.05 and ^∗∗^*P* < 0.01.

To explore the molecular mechanism underlying miRNA-181a in GC, caprin-1 was predicted bioinformatically as a putative target of miRNA-181a through TargetScan database (Figure 4A). To explore whether miRNA-181a targets caprin-1, dual luciferase assay was performed to determine the 3′-UTR of caprin-1 mRNA. The HEK293T cells transfected with miRNA-181a precursor and caprin-1-WT plasmid showed reduced luciferase activity in contrast to those transfected with the miRNA-181a precursor or caprin-1-Mut plasmid (Figure 4B). Furthermore, the caprin-1 mRNA and protein levels in the GC tissues were examined through qRT-PCR and Western blot analysis. As shown in Figures 4C,D, caprin-1 mRNA and protein levels were significantly lower in the GC tissues than in those in the adjacent non-tumor tissues (*P* < 0.01). IHC analysis of paraffin-embedded tissues revealed that the positive expression of caprin-1 was significantly lower in GC tissues compared with that in the adjacent non-tumor tissues [23.33% (21/90) vs. 66.67% (20/30)] (Figure 4E). We further verified the expression of miRNA-181a was inversely correlated with the expression of caprin-1 with Spearman’s correlation test (*n* = 90, *r* = -0.7284). The mRNA and protein levels of caprin-1 were determined after knocking down of miRNA-181a expression in the SGC7901 cells. We found that the protein level of caprin-1 increased, whereas the mRNA level of caprin-1 remained unchanged in the SGC7901 cells (Figures 4F,G). The caprin-1 protein level increased in the subcutaneous tumors of the xenografts (Figure 4H). These results demonstrated that miRNA-181a downregulates caprin-1 expression by binding to the caprin-1 3’-UTR.

### Caprin-1 siRNA Rescued miRNA-181a Oncogenic Effects in GC Cells

**FIGURE 5:**
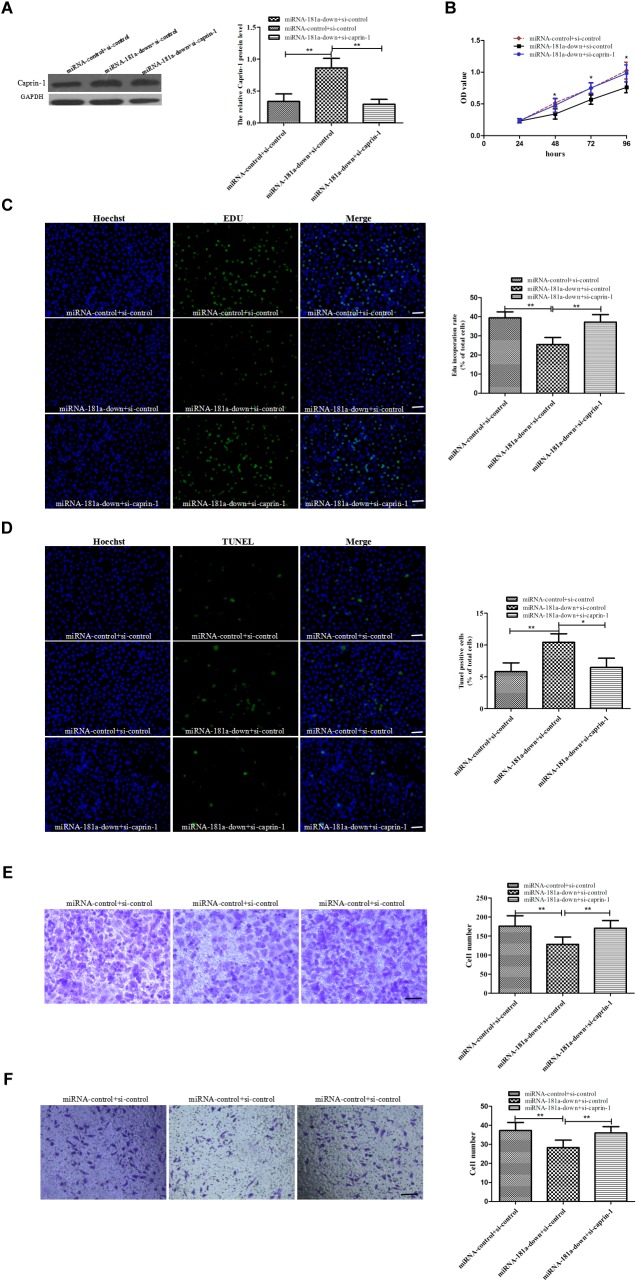
Caprin-1 silence rescues the oncogenic effect of miRNA-181a on GC cell **(A)** Caprin-1 expression decreased in the SGC7901 cells cotransfected with siRNA caprin-1 and miRNA-181a downregulated plasmid detected by Western blot analysis; **(B–C)** Cell proliferation was reversed in the co-transfected SGC7901 cells, as indicated by the CCK-8 (24, 48, 72, and 96 h) and EDU (48 h) assays, bar = 100 μm; **(D)** Cell apoptosis inhibited by EdUTP TUNEL Cell Detection in the co-transfected SGC7901 cells (48 h), bar = 100 μm; **(E–F)** Transwell migration and invasion detected in the co-transfected SGC7901 cells (48 h); ^∗^*P* < 0.05 and ^∗∗^*P* < 0.01.

To determine the role of caprin-1 in the oncogenic effects of miRNA-181a in GC, we performed rescue experiment. SGC7901 cells were co-transfected with miRNA-181a-knock-down plasmid or miRNA-181a-control plasmid and siRNA-caprin-1. It was found that the upregulation of Caprin-1 by miRNA-181a was counteracted by caprin-1 siRNA in GC cell as verified by Western blot analysis (Figure 5A). Cell proliferation, apoptosis, migration, and invasion assays were performed after cotransfection. As expected, caprin-1 siRNA reversed the oncogenic effects of miRNA-181a on GC cells (Figures 5B–F). These results demonstrated that the oncogenic effect of miRNA-181a was achieved at least in part by targeting caprin-1 in GC.

## Discussion

MiRNAs are involved in tumor initiation and metastasis (Chowdhury et al., 2018; Lin et al., 2018; Wang et al., 2018). MiRNA-181a has been identified as a multifaceted molecular regulator in different human tumors, and it may be a useful target for treatments of tumors (Wei et al., 2014; Zhang et al., 2014; Brockhausen et al., 2015; Zhu et al., 2016). Although miRNA-181a acts as a tumor suppressor in glioma (Shi et al., 2008), it is often regarded as an oncogene in other tumors, including neuroblastoma, hepatocellular carcinoma, breast cancer, and colorectal cancer (Schulte et al., 2010; Pichler et al., 2014).

In this study, we found that miRNA-181a was upregulated in GC tissues and in patients with lymph node metastasis, and the expression of miRNA-181a was correlated with tumor size, lymph node involvement, distant metastasis, and TNM stage. The cumulative survival rate of patients with high miRNA-181a expression was significantly lower than those with low miRNA-181a expression. These results showed that miRNA-181a was associated with the development and progression of human GC. Moreover, the expression of miRNA-181a was higher in a panel of GC cells compared to that of normal gastric epithelial cells. The highest expression of miRNA-181a was observed in the metastatic GC SGC7901 cells. Downregulation of miRNA-181a inhibited the growth, invasion, and migration but enhanced the apoptosis of the SGC7901 cells. Downregulation of miRNA-181a considerably inhibited tumor growth and metastasis in the mouse xenografts *in vivo*. These results suggested that miRNA-181a contributed to the development and progression of GC both *in vivo* and *in vitro.*

Caprin-1 is an RNA-binding protein that plays critical roles in human cancers. It promotes osteosarcoma tumor growth and lung metastasis in mice (Sabile et al., 2013) and regulates the proliferation and invasion of human breast cancer cells (Gong et al., 2013). Upregulation of caprin-1 is associated with poor prognosis in hepatocellular carcinoma (Tan et al., 2017). However, the expression patterns and biological functions of caprin-1 in GC have not been established.

MiRNAs control gene expression at the post-transcriptional and translational levels by binding to complementary sequences in the 3′-UTRs of target mRNAs (Li et al., 2018). We explored the underlying mechanisms and the correlations between miRNA-181a and caprin-1 in GC cells. Caprin-1 was predicted bioinformatically as a putative target of miRNA-181a. Dual luciferase assay demonstrated that miRNA-181a targets caprin-1 by binding to the 3′-UTRR of caprin-1 mRNA. MiRNA-181a expression was negatively correlated with caprin-1 expression in the GC tissues. Furthermore, the protein level of caprin-1 after knocking down of miRNA-181a in the SGC7901 cells showed a converse change with miRNA-181a, and caprin-1 knockdown reversed the oncogenic effect of miRNA-181a on GC cell proliferation, apoptosis, migration, and invasion. These results indicated that miRNA-181a functions as an oncogene in gastric tumorigenesis at least in part by targeting caprin-1 in GC.

Taken together, our data showed an interesting correlation between miRNA-181a and caprin-1 in GC. The upregulation of miRNA-181a and downregulation of caprin-1 were observed in the GC tissues and GC cell lines. MiRNA-181a enhanced the proliferation, migration, and invasion of GC cells and suppressed apoptosis by downregulating caprin-1, particularly by binding to the 3′UTR mRNA. MiRNA therapeutics is a growing field and has potential application in GC treatment. We highlighted the interaction between miRNA-181a and caprin-1 in GC development, which may provide a target for the exploration of novel therapeutic strategies for GC treatment.

## Author Contributions

QL and YC participated in most of the experiments, such as cell biology and molecular biology experiments. DS, YM, and KD performed CCK-8, Dual luciferase, EDU, and TUNEL incorporation assays. ML and YZ performed animal studies. SW and HL performed transwell assay. QL and DS directed data analysis. FZ designed the project.

## Conflict of Interest Statement

The authors declare that the research was conducted in the absence of any commercial or financial relationships that could be construed as a potential conflict of interest.
